# Viral modulation of synaptic pruning: implications for neuropathology and brain function

**DOI:** 10.1128/jvi.01684-25

**Published:** 2026-01-07

**Authors:** Shayan Aliakbari, Sareh Asadi, Mohammad Sayyah, Nima Naderi, Shakiba Salarvandian, Fariba Khodagholi, Hamid Gholami Pourbadie

**Affiliations:** 1 Neuroscience Research Center, Institute of Neuroscience and Cognition, Shahid Beheshti University of Medical Sciences 556492, Tehran, Iran; 2 Department of Physiology and Pharmacology, Pasteur Institute of Iran 89245, Tehran, Iran; 3 Neurobiology Research Center, Institute of Neuroscience and Cognition, Shahid Beheshti University of Medical Sciences 556492, Tehran, Iran; 4 Department of Pharmacology and Toxicology, School of Pharmacy, Shahid Beheshti University of Medical Sciences 556492, Tehran, Iran; 5 Sports Medicine Research Center, Neuroscience Institute, Tehran University of Medical Science 108844, Tehran, Iran; Indiana University Bloomington, Bloomington, Indiana, USA

**Keywords:** synaptic pruning, viral infection, synaptic density, complement cascade, microglia

## Abstract

Synaptic pruning is an essential neurodevelopmental process that refines neural circuits by eliminating superfluous or weak synapses, thereby enhancing cognitive functions, including learning and memory. Emerging evidence indicates that viral infections can profoundly influence synaptic processes throughout the nervous system. Viral pathogens have been shown to disrupt synaptic plasticity, alter synaptic protein expression, and dysregulate mechanisms responsible for synaptic elimination. These disruptions are often mediated through the activation of the complement system, inflammatory cytokines, and aberrant expression of postsynaptic density proteins. Depending on the nature and extent of infection, viral interference with synaptic pruning may result in either excessive synapse loss or synaptic retention, both of which are implicated in neuropathological outcomes, such as cognitive decline and neurodevelopmental disorders. This review examines the molecular and cellular mechanisms of synaptic pruning and highlights the impact of various neurotropic viruses on these processes. By elucidating the interplay between viral infections and synaptic pruning, we aim to provide insights into virus-associated neuropathology and inform future research directions and therapeutic strategies in the context of virology and neuroimmunology.

## INTRODUCTION

During development, the excess of synapses needs to be eliminated to regulate their number and function. Synaptic pruning is a crucial neurodevelopmental process in which microglial cells engulf and eliminate “weak” or inactive synapses (1). This mechanism ensures that neural circuits are efficiently refined, enhancing cognitive functions and overall brain health. The phenomenon of synaptic pruning was first observed in the 1970s within the fetal cat’s dorsolateral geniculate nucleus (dLGN) (2). Since then, extensive research has demonstrated that synaptic pruning occurs in different regions of the brain, such as the amygdala (3), cerebellum (4), prefrontal cortex (5), olfactory bulb (OB) (6), and hippocampus (7). This process plays a pivotal role during early developmental stages, facilitating the proper maturation of neural networks and contributing to the formation of a functional and efficient brain. The understanding of synaptic pruning has significant implications for neuroscience, providing insights into the mechanisms of neural plasticity and the maintenance of cognitive functions throughout the lifespan. Synaptic pruning may also remove small segments of axonal and dendritic spines in presynaptic and postsynaptic terminals in the CNS of vertebrates and invertebrates. From mid-embryonic development to adolescence, synaptic pruning refines neuronal connections in the brain, such as the corpus callosum (8).

Downregulation of synaptic pruning in the nervous system contributes to aberrant connections that are linked with neuronal diseases such as autism (9), epilepsy (10), and schizophrenia (11). On the other hand, multiple studies have shown negative effects of complement system-regulated synaptic pruning in neurodegenerative disorders, including Alzheimer’s disease (AD), Huntington’s disease, multiple sclerosis (MS), and multiple system atrophy (12), and Parkinson’s disease (PD), which leads to a massive elimination of synapses and contributes to cognitive and behavioral impairment (13). In this line, synaptic dysfunctions in the medial prefrontal cortex (mPFC) in mice have contributed to significant reductions in the elimination of synapses and disruptions in both functional and structural connectivity. These alterations are associated with neuropsychiatric disorders (14).

Infectious agents, including viruses, have been linked to functional and structural changes that affect synaptic plasticity and synaptic pruning. It has been shown that viral infections, such as influenza, rabies, borna, herpes simplex, coronavirus disease 2019 (COVID-19), and human immunodeficiency virus (HIV) (4), alter synaptic transmission and contribute to aberrant synaptic pruning in the brain through alteration of gene expression, synaptic proteins, and regulation of proinflammatory cytokines (13, 15).

## MECHANISMS OF SYNAPTIC PRUNING

### Microglial phagocytosis

Microglia are responsible for synaptic remodeling and synaptic refinement in the CNS. Research has shown that the fractalkine and Triggering Receptors Expressed on Myeloid cells 2 (TREM2) signaling pathways are involved in synaptic pruning within the hippocampus (16, 17). C-X3-C Motif Chemokine Ligand 1 (CX3CL1), as a Fractalkine receptor ligand, is a distinctive chemokine that exists in membrane-bound forms, playing a crucial role in neuro-glia communication and modulating microglial activity. It plays an important role in synaptic pruning within the cerebellum (18, 19). CX3CL1 levels increase in the hippocampus following pilocarpine-induced status epilepticus, a model of temporal lobe epilepsy (TLE) (20).

Activation of the complement pathway in the CNS also promotes microglia-regulated synaptic pruning during neurodevelopment (21). In this regard, hippocampal sclerosis in TLE patients contributes to an increase in the complement compartments that affect seizure pathophysiology. In models of absence seizures in the somatosensory cortex, it has been well indicated that knockout of complement component 1q (C1q) in mice leads to increased spine density, synaptic connectivity, and excitability (22). Conversely, increases in the level of C1q are associated with synaptic pruning in models of neurodegenerative disorders (13). Another group showed that C1q knockout leads to the accumulation and deposition of phosphatidylserine (PS) of presynaptic structures in the retinogeniculate system (23). Additionally, Complement component 3 (C3), as one of the proteins of the classical complement pathway, mediates synaptic pruning during development (24). Among the number of complement molecules, C3 is activated in both classical and lectin pathways, controlling the elimination of synapses during brain development (25). Alternative pathways can be directly initiated from C3b. C3b activates the effector pathways of the complement system linked to Complement 3a receptor (C3aR). Complement proteins C1q, C3, and C4 mediate synapse elimination by tagging inappropriate neuronal connections for removal. During the synaptic pruning period, microglia transition to a specialized, highly phagocytic state to execute this process (26). Studies with C1q and C3 knockout mice showed abnormal synaptic activity that may be due to a disruption of synaptic loss (11).

**Fig 1:**
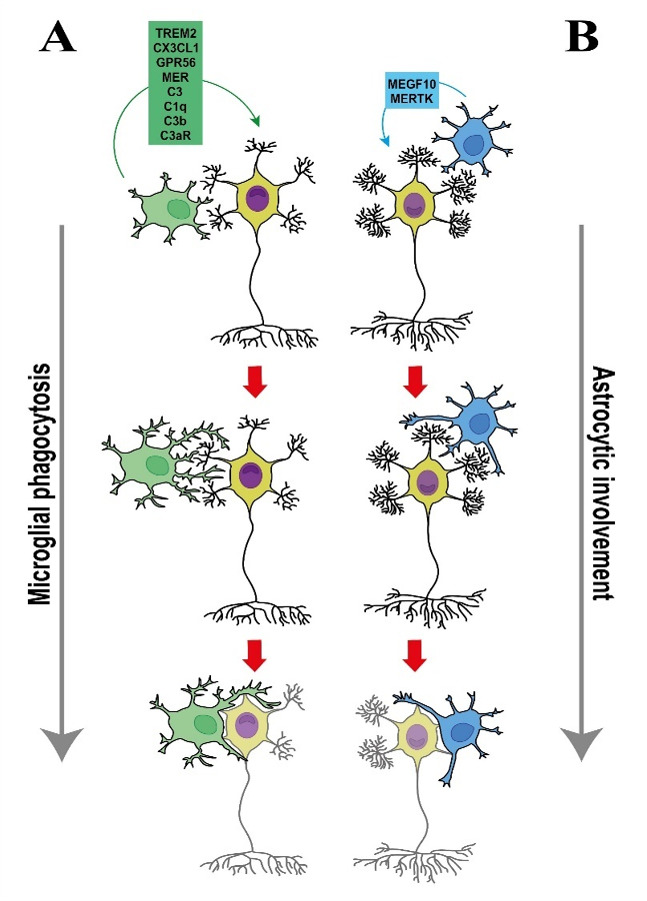
Microglial phagocytosis (**A**) and astrocyte involvement (**B**). It has been indicated that TREM2, CX3CL1, GPR56, MER, C3, C1q, C3b, and C3aR regulate synaptic refinement in both cortex and hippocampus neurons associated with synaptic pruning. TREM2, through interaction with complement systems including C3, C3b, and C3aR, prunes unnecessary synapses (**A**). MEGF10 and MERTK promote synaptic pruning in the CNS. Loss of MEGF10 and MERTK in the hippocampus, thalamocortical, and dLGN prevents synaptic elimination (**B**).

In addition to the pathways mentioned above, several other mechanisms contribute to the synaptic pruning processes performed by microglia (Fig. 1). In the hippocampal neurons and microglial culture, activation by PS mediates synapse pruning (27). A mutation in the TREM2 also plays a central role in promoting neurodegenerative disorders and behavioral dysfunctions (28). TREM2 is significantly expressed in the hippocampus at early stages of neuronal progression, and TREM2 knockout affects microglia activation in the CNS in both the cortex and hippocampus (17). A study recently reported that a mutation in TREM2 leads to an increase in synapse density, spine elimination, and spine refinement (17). Deficiency in TREM2 expression results in disrupted synaptic engulfment in hippocampal neurons (29). It has been suggested that TREM2 restricts synaptic loss in the early stages of hippocampal development. Loss of TREM2 leads to excessive synaptic pruning by astrocytes during hippocampal development (29). Reports have also shown that TREM2 can regulate synaptic pruning by adding C1q and C3 proteins in the retinogeniculate pathway (30). Additionally, TREM2 can promote the pruning of inactive synapses in the cortical prelimbic area (31). TREM2, in AD, promotes synaptic clearance via interaction with Apolipoprotein E (APOE) and C1q (32). It was also suggested that TREM2 may regulate synaptic pruning via binding to PS (17). PS using milk fat globule-EGF-factor 8 (MFG-E8) elevates the elimination of dendritic spines in both the OB and hippocampus and disrupts the maturation of the synapses in the hippocampus, such as dentate gyrus (DG) (6). Furthermore, the pathways mediated by Mer receptor tyrosine kinase (MER) and G protein-coupled receptor 56 (GPR56) are also crucial for regulating synaptic pruning (33), particularly in inhibitory post-synapses. These receptors interact with PS located on the surface of apoptotic cells and control synaptic remodeling (23, 34). MER, through the clearance of apoptotic cells, binds to PS and mediates the phagocytic removal of unnecessary or damaged synapses. Similarly, GPR56, another receptor that interacts with PS, participates in the regulation of synaptic pruning by influencing microglial activity and eliminating specific synaptic components (Fig. 1).

### Astrocytic involvement

Astrocytes, in the CNS, regulate synapse formation, function, and elimination. It has been discovered that the phagocytic activity of astrocytes in synapse elimination is critically dependent on two key phagocytic receptors: multiple EGF-like domains 10 (MEGF10) and Mer Tyrosine Kinase (MERTK) (Fig. 1). MEGF10 is a cell surface receptor known for its role in mediating the engulfment of synaptic debris by astrocytes (35). It binds to synaptic elements that need to be removed, facilitating their uptake and clearance. This receptor is crucial for ensuring the efficient removal of excess or damaged synapses, which is critical for preserving proper neural connectivity and function (33).

Similarly, MERTK, another important receptor, is involved in the phagocytosis of synaptic components. MERTK interacts with PS, which is exposed on the surface of dying or remodeling synapses, to promote their recognition and ingestion by astrocytes. This interaction is vital for the orderly pruning of synaptic connections in response to neuronal activity. MEGF10 and MERTK are also involved in neural activity-dependent synapse pruning. Studies show that a deficiency in both MEGF10 and MERTK can lead to disruption in synapse elimination in dLGN, as a classical model of developmental synapse elimination (36). Thus, high expression of MEGF10 and MERTK in the dLGN can promote synaptic scaling. Also, MEGF10 mutation in astrocytes has been shown to impair synapse pruning and the reduction of synapse number in the thalamocortical network and V_1_ after monocular deprivation in mice. In addition, MEGF10 deletion in hippocampal Cornu Ammonis (CA1) neurons impairs synaptic plasticity and memory formation (33), probably due to disrupted clearance of weaker/deprived synapses by astrocytes.

### Neuronal activity

Neuronal activity is a fundamental mechanism that regulates synaptic strength and drives the reorganization of neural circuits (37). The neuronal activity results in expression of early genes, host genes that are upregulated in response to neuronal activity, which triggers the elimination of neural circuits. Several genes, such as Arc and JAK/STAT, influenced by a specific pathway, affect synaptic arborization in response to intense neuronal activity (38). Specifically, the Arc protein in the Purkinje cell of the cerebellum regulates the pruning of the redundant synapses (39). Also, it has been shown that high expression of Arc induced by myocyte enhancer factor 2 mediates synaptic loss in hippocampal neurons (39). In addition, Arc is required in the induction of long-term depression (LTD) and cell-wide synaptic scaling in inactive spines via α-amino-3-hydroxy-5-methyl-4-isoxazole propionic acid (AMPA) receptor endocytosis (40). During development, JAK2/STAT1 signaling plays a remarkable role in the refinement of synapses in both the cingulate cortex and hippocampus.

## GUIDANCE MOLECULES IN NEURAL PRUNING

### Neurotrophic factors

Brain-derived neurotrophic factor (BDNF) in the brain serves a key role in synaptic plasticity and synaptic pruning. BDNF mediates a role in cognitive functions and synaptic connections. For instance, BDNF-mediated synaptic plasticity and enhanced neuronal autophagy contributed to the reinforcement of synaptic pruning in the brain (41, 42). Orefice et al. showed that mature BDNF regulates spine pruning through activating Rac1 and the TrkB pathway (43). Pro-BDNF activates RhoA via the p75NTR receptor, which fosters spine growth at active synapses (43). In contrast, mature BDNF utilizes the same receptor to directly induce axon degeneration during the process of developmental pruning (44). The physiological mechanism of synaptic pruning by BDNF has not been completely found. It has been suggested that a disruption in pro-BDNF/mature BDNF may create synaptic elimination in cognitive disorders. For example, Yesilkaya et al. reported that an imbalance in pro-BDNF/mature BDNF is linked to the progression of schizophrenia, as it is hypothesized that schizophrenia may be associated with excess synaptic pruning (45).

### Semaphorins

Semaphorins are conserved proteins that are expressed widely throughout the CNS as well as various aspects of nervous system development, including neuronal morphogenesis, synaptic plasticity, electrical activity, synaptogenesis, neurogenesis, and synaptic refinement (46). It has been recently shown that members of the Semaphorins can play essential roles in neural connections and regulate synaptic functions. Among all the Semaphorins, Class 3, including Sema3A, Sema3F, Sema3E, and Class 4 secreted Semaphorins such as Sema4B and Sema4D, and Sema6A, Sema5A, and Sema5B, can directly or indirectly regulate dendritic development from embryogenesis to adulthood in the hippocampus, medulla, OB, retina, and basal ganglia (47–49). Among all members of semaphorins, Sema3A and Sema3F have the most important roles in synaptic pruning. For example, during postnatal mouse development, Sema3A through Npn-1/PlexA4 signaling promotes dendritic arborization, synapse distribution, and neuronal polarity in the layer 5 pyramidal cells of the cortex (50). Other studies have indicated that Sema3A and Sema3F can trigger the pruning of hippocampal neurons by PlexA3 (51). Furthermore, in the granule cells (GC) of the hippocampus, Sema3A, via activating tyrosine phosphorylation (Tyr 397) and serine phosphorylation (Ser 732) of FAK, can regulate synapse formation by modulating the neurite branching of selected neurons (52). In cortical neurons, Sema3F activates two signaling pathways involving small GTPases Rac1 (Rac1-PAK1-3-LIMK1/2-Cofilin1) and Rho-A (RhoA-ROCK1/2-Myosin II), leading to a significant reduction of dendritic spines (53). Also, Sema3F via Tiam1 and Rac1 fosters synapse elimination (54). Moreover, it was reported that Sema3A mediates axon repulsion via Npn1 and PlexA2 activation (55).

### Netrins and their receptors (DCC and UNC-5)

Netrins, secreted axon guidance proteins, regulate synaptogenesis, synaptic plasticity, dendritic growth, and synaptic elimination in the CNS through regulating the Rho-GTPases Cdc42, RhoA, and Rac1, enhancing intracellular Ca^2+^ (56). In the CNS of mammals, DCC and UNC-5, two classes of Netrin receptors, can directly mediate axon migration, morphogenesis, and axonal repulsion during brain development, but the mechanisms are not completely uncovered. It has been shown that DCC is involved in both attractive and repulsive responses, but UNC5 can only regulate axonal repulsive responses (57). Furthermore, DCC in the hippocampus of mature mammals can regulate the dendritic arborization of nascent synapses in pyramidal neurons (58). In addition, it has been indicated that in the retinal ganglion cell of the Xenopus, DCC can directly influence dendritic growth without altering branch numbers, whereas UNC-5 significantly increases new branches with decreased branch stabilization (59). Also, UNC-5 leads neurite growth and regulates axonal remodeling in motor neurons in the neuroblastoma cells of *C. elegans* (60). Thus, DCC and UNC-5 mediate axon guidance, maintenance, and dendritic arbor differentiation.

### Ephrin and ephrin receptors

The ephrin receptor family, transmembrane tyrosine kinase receptors, is required in the regulation of neuron morphology. Furthermore, ephrin and ephrin receptors are implicated in various functions in the brain, such as emotion, learning, memory, synaptogenesis, synaptic pruning, synaptic transmission, fear conditioning, memory formation, and associative memory (61). Research has shown that deletion of ephrin in the hippocampus leads to impairment in synaptic formation and remodeling, which are linked to difficulties in recalling contextual memory (62). In the same direction, Nguyen et al. showed that deletion in the CA_1_ hippocampal neurons in mice can potentially lead to a reduced formation of new spines, which can impair the recall of contextual memory (62). Mice with a lack of ephrin-B (EB) receptors exhibit abnormal spine morphology in the hippocampus pyramidal neurons (63). However, Koeppen et al. demonstrated that ephrin B_1_ receptor deletion in the hippocampal CA_1_ region of male mice caused an elevation in immature dendritic spines and enhanced contextual fear memory recall (64). It has been shown that during postnatal development, ephrin-B3 (EB3), a type of Ephrin receptor, can mediate the elimination of hippocampal mossy fiber axons in mice (65). Also, the postsynaptic EB3 receptor, in the Schaffer/CA_3_-CA_1_ circuit, is required for dendrite engulfment and spine formation (66). It is in line with previous studies showing that EB3 in neurons of the cortex and hippocampus can play an essential role in axon pruning during postnatal development (65, 67). Also, EB3, in neurons of the basolateral amygdala, mediates axon pruning required for innate fear behavior (68). These findings exhibit that Ephrin receptors play a mediatory role in dendritic spine development.

## VIRAL INFECTION

Viral infections can have profound impacts on the nervous system, often leading to synaptic dysfunction by inducing apoptosis and damaging the blood-brain barrier (69). One of the critical processes affected is synaptic pruning (70), which is important for the development of healthy neural networks. A virus in the CNS leads to an alteration in brain function (71). These neurotropic viruses affect synaptic activity and cause behavioral diseases, such as anxiety (72), aggression (73), and social isolation (74) that are linked to the turnover of dendritic spines (71). Infection of neurons, astrocytes, and microglia by different viruses can induce apoptotic pathways and upregulate the secretion of pro-inflammatory cytokines, including interleukin-1 beta (IL-1β), 8, and tumor necrosis factor (TNF-α) (75). The response caused during a viral infection can inadvertently target synapses, leading to their removal or dysfunction (Table 1). This can destroy the delicate balance of synaptic connections, resulting in cognitive and neurological impairments. Understanding the mechanisms by which viral infections influence synaptic pruning is crucial for developing therapeutic strategies to mitigate these adverse effects and preserve neural function.

**TABLE 1:** Common human viral infections are involved in the elimination of synapses

Major viral family	Strain(s)	Host(s)	Effect on neuronal function	Mechanism
Flaviviridae	West Nile virus and Zika virus	Monkey/cat/mosquito	Synaptic impairment/neuronal damage (76)	Via inflammatory mediators such as TNF-α, IFN-β, IFN-γ, IL-6, IL-1β, and complement systems, synaptic elimination
Retroviridae	Human immunodeficiency virus	Common chimpanzee	Loss of excitatory synaptic connections and mild neurocognitive impairment (77)	GluN2B subunit of the NMDA receptor increased the high influx of calcium
Rhabdoviridae	Rabies virus	Dog/bat/rodents	Dysfunction in synaptic transmission via decreased neurotransmitter release (78)	Depolymerization of actin filaments (F-actin)
Coronaviridae	Coronavirus	Birds/mammals	Olfactory dysfunction and stroke (79)	Tyrosine kinase signaling pathways, JAK/STAT signaling, and the TLR4 pathway
Orthomyxoviridae	Influenza A	Birds/mammals	Disbalance in glutamatergic synaptic transmission (80)	Secretion of inflammatory markers such as IL-1β
Bornaviridae	Borna virus	Birds/mammals	Abnormal synaptic plasticity (81)	Internalization of AMPA receptors and AChRs activated by PKC
Herpesviridae	Herpes simplex virus	Mammals	Changing the expression of Arc and CaMKIIb regulates LTP and memory consolidation (82)	Expression of CREB and Arc protein and release of inflammatory markers, including IFN-β, IFN-γ, TNFα, IL-1β, IL-6, chemokine (C–X–C motif) ligand 8 (CXCL8) and TGFβ

## SYNAPTIC PRUNING IN VIRAL INFECTION

It has been suggested that during viral infections, the activation of glial cells leads to synaptic pruning as part of the pathogenesis (70). Several studies have shown that during CNS infection, activated microglia promote synaptic loss through increased production of proinflammatory cytokines and chemokines (83–85). Murine models of brain infection also indicated that interferon signaling activated by CD8^+^ T cells in microglia exacerbates synapse elimination in viral infections (84). Furthermore, following viral infection, high accumulation of protein in the endoplasmic reticulum (ER) initiates the degradation of the antioxidant cellular systems, possibly modifying synaptic elimination (86). For example, in COVID-19 patients, neuronal cell death-mediated synaptic pruning is due to the changes in the antioxidant cellular system (84). The key role of viral infections in synaptic pruning across different regions of the brain is reviewed below.

### Zika virus

**Fig 2:**
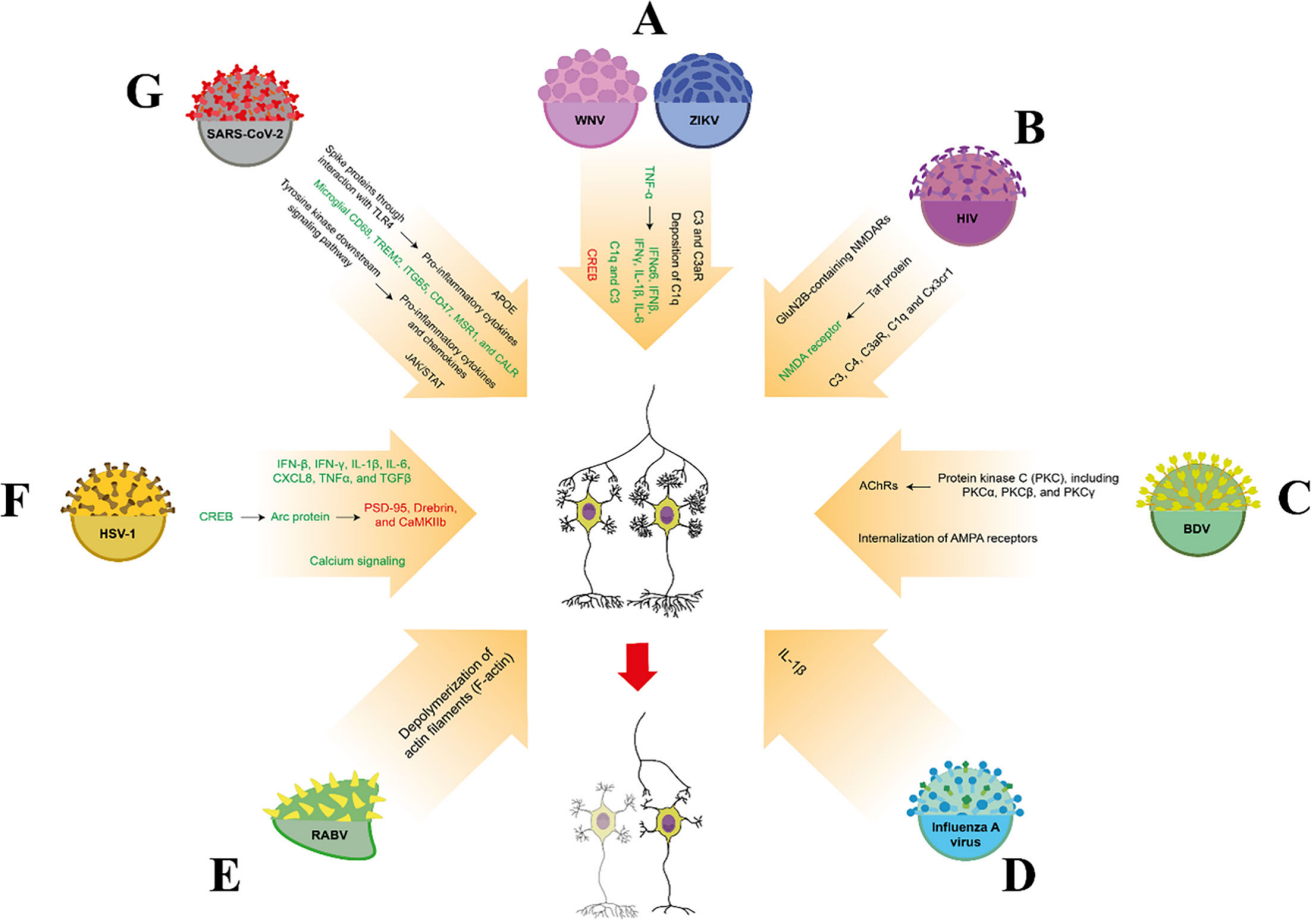
Major role of the virus in synaptic pruning. West Nile virus (WNV) and ZIKV increase neuroinflammation in the CNS. Neuroinflammation elevates inflammatory mediators such as TNF-α, IFN-β, IFN-γ, IL-6, and IL-1β-induced synaptic elimination in various brain regions. Also, infection of these viruses can alter synaptic remodeling via high expression of complement proteins (**A**). HIV infection can promote the GluN2B subunit of the NMDA receptor-initiated a high influx of calcium, changing the elimination of synapses (**B**). It has been indicated that BVD infection regulates synaptic pruning through two pathways: AChRs activated by PKC-induced LTD in the CNS, such as the hippocampus and cortex, and internalization of AMPA receptors during induction of the LTD (**C**). Influenza viruses, including H1N1, augment synaptic elimination via the release of inflammatory markers such as IL-1β (**D**). Rabies virus, with the depolymerization of actin filaments (F-actin), increases synaptic pruning in the CNS, including dendritic spines of the CA1 region (**E**). The infection of the HSV-1 augments the expression of the CREB-induced Arc protein. Arc protein also limits PSD-95, Drebrin, and CaMKIIb-modified synaptic scaling. Also, HSV-1 infection via an increase in intracellular calcium level and inflammatory mediators such as IFN-β, IFN-γ, IL-1β, IL-6, chemokine (C–X–C motif) ligand 8 (CXCL8), TNFα, and TGFβ causes synaptic pruning (**F**). SARS-CoV-2 exposure triggers the expression of CD68, TREM2, ITGB5, CD47, MSR, and CALR genes involved in synaptic elimination. Also, COVID-19 infection, with the initiation of tyrosine kinase signaling pathways and JAK/STAT signaling, increased cytokines and chemokines and played a key role in the refinement and elimination of synapses in the brain. Furthermore, this virus can, via APOE and TLR4 expression, regulate synaptic loss in multiple brain regions (**G**).

The family of Flavivirus, single-stranded RNA viruses, neurotropic, neuroinvasive, positive, and enveloped RNA viruses, is transmitted by insects, including mosquitoes and ticks, and can infect humans. The family of Flavivirus consists of several viruses, such as the Zika virus (ZIKV). These viruses play a noticeable role in the immune responses and apoptosis (87). The ZIKV was first identified in Uganda in 1947 and has since been found to cause significant cellular and molecular alterations in neural cells during both pregnancy and adulthood (88). ZIKV leads to neurological disorders in humans, including encephalopathy and behavioral dysfunction (89). Chronic ZIKV triggers neuroinflammation in the subregions of the hippocampus, the brain regions critical for memory formation (85). Interestingly, the ZIKV targets the hippocampus, causing impairment in synaptic plasticity (Fig. 2). In cultured mice, the subventricular zone of the anterior forebrain and the sub-granular zone of the hippocampal slices, chronic ZIKV exposure affects neural stem cells from the hippocampus in adult rats, correlated with neuronal apoptosis, synaptic impairment, and reduced proliferation (90). Experimental models also indicated that ZIKV exposure can change neuronal communication (76). Alteration of glia-neuron communication by the ZIKV is dependent on the expression of molecular signaling cascades in the brain. ZIKV through nuclear factor erythroid 2-related factors 2 (Nrf2) and nuclear factor κB (NF-κB) signaling cascades provoked neuroinflammation in the DG of the hippocampus associated with neurodegenerative diseases (76). Expression loss of Nrf2 in the hippocampus is associated with astrogliosis and inflammatory mechanisms (76). Activation of Nrf2 due to ZIKV acute infection in the CNS produces several antioxidant systems, such as Glutathione (GSH), glutamate cysteine ligase catalytic subunit (GCLC), and glucose 6-phosphate dehydrogenase (G6PD), but the inhibition of these antioxidants increases viral replication (86). In the hippocampus, NF-κB may be an essential component in ZIKV-induced cellular damage in the brain by regulating the expression of cytokines and chemokines. NF-κB in the brain can activate STING (stimulator of interferon genes) upon ZIKV infections (91). Also, in response to ZIKV infection, NF-κB increases proinflammatory cytokines such as IL-1β and IL-6 (92) and mediates synaptic loss. Recently, it has been indicated that many flaviviruses, including ZIKV, through high expression of IFN-γ, are involved in the elimination of synapses and axons. IFN-γ leads to the engulfment of neurons, reduction of neuronal apoptosis, and loss of postsynaptic terminals in animals infected by the ZIKV in the subregions of the hippocampus (85). IFN-γ plays a fundamental role in synapse formation (93), memory, and learning (94). Mice with loss of IFN-γ indicate elevated neurogenesis, promoted hippocampal plasticity, enhanced cognitive performance, and synapse formation (95). Loss of IFN-γ showed high rates of synapse configurations in the hippocampal neurons, cerebellum, and the prefrontal cortex (PFC), the brain regions that are involved in learning and memory, movement, and decision-making, respectively (95). IFN-γ attaches to nerve cells, setting off a chain reaction inside the cell. That leads to changes that destroy synapses. For this destruction to occur, phosphorylation of a protein called STAT1 occurs by enzymes called JAK1 and JAK2, which play a key role in synapse stripping (96). Neuronal apoptosis in CA_1_ pyramidal neurons in mice with ZIKV infection can affect spatial learning (97).

ZIKV in the CNS induces TNF-α that affects neuronal signaling, neuronal differentiation, and synaptic plasticity (98–100). Figueiredo et al. report that in temporal lobe cortical slices 48–72 h after infection, ZIKV leads to the increased level of TNF-α, overexpression of C1q/C3, brain inflammation, and memory dysfunction (14–30 after infection) in mice (101). Blocking of TNF-α signaling and complement molecules such as C1q/C3 restores learning and memory deficits in these mice. Together, these findings confirm that cytokine and complement molecules are activated in the CNS following ZIKV infection. It has also been shown that ZIKV can increase C1q, C3, and its receptor in the CNS. ZIKV infection can initiate the signaling of the complement systems associated with synaptic remodeling and memory impairment in mice (101). Figueiredo et al. also reported that phagocytosis of hippocampal synapses infected by ZIKV can act via complement systems such as C1q/C3 in parietal cortex (101). Human studies have shown that acute infection with ZIKV, neural progenitor cells (NPC) play an important role in synaptic pruning through interaction with the complement system in the subventricular zone (102). The dysfunction in NPC signaling can create abnormal synaptic pruning in neurodevelopmental disorders, including autism and schizophrenia. CX3CL1-CX3CR1, through NPC signaling, maintains homeostatic pruning of synapses. Disruption of this signaling leads to dysfunction in synaptic pruning (103). Thus, these findings exhibited that ZIKV triggered neuroinflammation, phagocytosis of hippocampal neurons, and cognitive impairments in mice related to the elimination of synapses. Overall, ZIKV infection alters synaptic pruning through both neuronal and immune-mediated mechanisms, leading to cognitive impairment (Fig. 2). This highlights the importance of understanding the mechanisms behind ZIKV-induced neural damage, resulting in the development of therapeutic strategies to mitigate its effects.

In summary, ZIKV initiates multifaceted inflammatory responses, thereby disrupting synaptic homeostasis. The virus triggers high levels of inflammatory proteins, including TNF-α, IFN-γ, and IL-1β, centered on activation of the NF-κB cascade, which, in turn, activates the classical complement cascade (C1q/C3). This immune response leads to opsonization of synapses and their removal by phagocytosing microglia. Among flaviviruses, this pathway indicates a common strategy in which host antiviral defenses are hijacked to cause damage to neural circuits and cognitive deficits.

The mechanism of synaptic pruning by ZIKV, which is fundamentally dependent on activation of the complement system in the course of neuroinflammation, places it in a position close to other neurotropic flaviviruses, such as WNV (discussed below). In contrast, viruses such as HIV induce complement-independent synaptic elimination via proteins like gp120 and Tat.

### West Nile virus

WNV, a neurotropic flavivirus, is transmitted by mosquitoes and leads to encephalitis and paralysis in humans and birds (104). Infection with WNV causes changes in glial cells. These alterations are linked with the activation of pro-inflammatory markers such as IL-1β in the hippocampal CA_3_, which creates inhibition of neuronal repair (105, 106). Animal studies showed that following 10- and 26-day post-infection in neurons of the hippocampus, WNV induces spatial and contextual memory impairments (107). Also, patients with chronic infection of WNV exhibit visuospatial processing and memory impairments (108, 109). Chronic infection by WNV has been shown to induce the elimination of presynaptic terminals, the specialized end of a neuron’s axon (85). Notably, the elimination of presynaptic terminals in mice infected by WNV is dependent on the complement molecules, such as C3 and its receptor C3aR (106). Additionally, WNV is linked to synaptic loss similar to that seen in AD, primarily due to the deposition of complement component C1q at synapses (110). As mentioned earlier, the complement compartments play an essential role in the activation of microglia and the subsequent synaptic pruning. In addition, infection with WNV triggers defects in the synaptic terminals of hippocampal CA_3_ (106).

These findings suggest that the expression of complement systems in the hippocampus leads to synaptic terminal loss, which is associated with learning and memory defects. Notably, over 50% of these cases are caused by WNV (106). Similarly, Vasek, M. J. et al. demonstrated that WNV infection in mice activates complement proteins, such as C1q/C3, which target synaptic pruning in the CNS. However, it has been shown that mice with disrupted expression of IL-34, C3, or C3a receptors do not experience neuronal loss (106). In addition to ZIKV infection, in patients with chronic WNV infection, the TNF-α signaling cascade is initiated, which is correlated with inflammation (111). In response to WNV infection, TNF-α, a major inflammatory cytokine, directly impairs the function of brain organoids (miniature brain models).

TNF-α activates caspase-8 and caspase-3 cascades involved in the presynaptic axon elimination. During synapse elimination at the neuromuscular junction, Caspase-3 activity in the motor neuron leads to a decrease in βIII-Tubulin, a marker of neuronal maturity (112). Since TNF-α is a known inducer of synaptic pruning by microglia (113), it provides strong evidence that this inflammatory pathway leads to neuronal injury and possibly synapse loss.

The pathogenesis of WNV highlights the role of the complement system as a major architect of synaptic pruning in flavivirus-induced encephalitis. Viral infection leads to the high deposition of C1q and C3 at synapses, facilitating their clearance through microglia. This process is strengthened by a potent inflammatory environment mediated by TNF-α, which can directly initiate pro-apoptotic signaling in neurons. The remarkable mechanistic overlap between WNV and ZIKV reveals a common pathogenic strategy in this virus family, suggesting that targeting the complement-inflammation axis could be an effective therapeutic approach.

WNV and ZIKV, as members of the Flaviviridae family, share a similar pattern of synaptic pruning that is strongly dependent on C1q/C3 signaling pathways. However, WNV infection is also strongly associated with TNF-α-mediated activation of caspase cascades. This direct apoptotic pathway, which may contribute to synaptic loss, could distinguish WNV’s long-term pathology from that of ZIKV.

### Human immunodeficiency virus

Significant studies have also been conducted on the key role of immunodeficiency virus type 1 (4) in synaptic pruning. HIV, as a neurotropic virus, also impacts synaptic plasticity. HIV in approximately 50% of individuals can create cognitive and behavioral impairments (114). HIV-1 frequently leads to cognitive and behavioral deficits associated with neurocognitive disorders. In the CNS, this behavioral dysfunction is related to HIV proteins such as Tat. Tat injection promotes activation of the N-methyl-D-aspartate receptors (NMDAs, channels permeable to Ca^2+^), Ca^2+^ influx (115), and dysfunction in mitochondrial metabolism and proteasome in the spinal cord dorsal horn of HIV patients (116). Research indicates that the HIV Tat protein mediates the toxic effects of infected brain cells, leading to neuronal apoptosis and impairments in synaptic and dendritic function (117). This protein in glial cells is associated with neurological disorders by promoting mechanisms including increased oxidative stress, neuroinflammation, and disrupted neuronal signaling, ultimately leading to decreased neuronal excitability and impaired learning and memory (118). These inflammatory markers, such as IFNs, TNFα, and ILs, can change synaptic transmission associated with long-term potentiation (LTP; LTP is the process by which the connection between two neurons is strengthened through repeated, simultaneous activation, and it is believed to be a cellular basis for learning and memory) in CA_1_ hippocampal neurons (119). Furthermore, these neuro-immune mediators can lead to cognitive deficits correlated with loss of synaptic connections (116) and loss of glutamatergic synapses (77) that affect scaling synapses induced by HIV Tat. HIV induces hippocampal synaptodendritic injury and subsequent memory impairment in the immunocompetent mouse brain through a previously unrecognized, non-neurotoxic viral function (120). Furthermore, 7 or 28 days after infection, HIV leads to synapse loss and neuroinflammation in the CNS, which is related to the high expression of classical complement cascade components, such as C1q and C3 (121). C1q is activated by several factors, such as RXRα, RAR, and LXRα (122). Although C3 is induced by HIV Tat downstream of NF-κB and IL-6 (123). It has been shown that C1qa knockout mice are not protected from Tat protein-created synaptic loss in the cortex (121). Thus, C1q regulates the synaptic loss in the CNS of HIV patients (121). In addition to C1q, it has been recently shown that C3, C4, C3aR, and CR3 can lead to synapse elimination through phagocytic microglia in different disease models (124). Also, neuroinflammation induced by Tat injection can increase high levels of C1q and C3 in mice associated with synaptic elimination. However, Hammond et al. reported that C1q is not involved in the synaptic loss contributed to HIV-1 Tat (121). It has been reported that HIV proteins, such as Tat or gp120, induce the high degradation of synaptic markers in transgenic animals via the activation of the NMDA receptor and elevate a high influx of Ca^2+^ via this receptor (77). Hargus et al. indicated that HIV potentiates GluN2B-containing NMDARs (a specific subtype of NMDA receptors) and regulates pro-apoptotic pathways and PERK/eIF2α (protein kinase RNA-like ER kinase/Eukaryotic Initiation Factor 2 alpha) pathway (125) that affects synaptic scaling (126). Aksenov et al. also indicated that a single injection of Tat caused synapse loss and neuroinflammation 7 days later in the striatum (127). Kim et al. also reported that glycoprotein gp120 and HIV proteins lead to the elimination of synapses in animal models 24 h after infection (128). Furthermore, the accumulation of the HIV protein gp120 in postmortem patient tissue has been linked to altered synaptic efficacy and is implicated in the elevated pain perception associated with HIV infection (129). Ru et al. reported that in spinal pain, chronic infection with HIV, gp120 induces synaptic loss through the Wnt/β-catenin/FKN/CX3R1-specific signaling pathway (130), so that the inhibition of this pathway blocks gp120-induced synaptic loss. The Wnt/PCP pathway is required in the JNK activation, intracellular Ca^2+^ concentration regulation, and protein kinase C (PKC) activation in HIV-induced neuropathic pain (131). It is shown that Wnt5 signaling through interaction with JNK is involved in sensory dendrite pruning (132). Thus, this signaling pathway may be involved in synaptic elimination. Also, this protein can decrease the expression of synaptic markers such as postsynaptic density protein 95 (PSD-95); a major scaffolding protein in the postsynaptic terminal of excitatory synapses and Syn I, which mediate synaptic degeneration in cortical neurons (130). HIV gp120 stimulates abnormal levels of nitric oxide (NO; a retrograde neurotransmitter in synapses) in the cortex due to high entry of Ca^2+^ via NMDA receptor, which is implicated in the mechanism of neuronal death (133). The neuronal death can create an abnormal balance between the inhibitory and the excitatory units and alter stabilized synaptic pruning (134). Sa et al. reported that HIV-1 infection can lead to a decrease in spine density in the hippocampus related to cognitive decline (135, 136). According to McLaurin et al., in transgenic rats, HIV-1 causes a disruption in synaptic pruning, synaptic connectivity, and synaptic efficacy in the mPFC (137). The mPFC and hippocampus from deceased AIDS patients have shown that a significant reduction in key synaptic markers, such as PSD-95 and synaptophysin, has a direct effect on synaptic density, which is associated with the development of symptoms of neurocognitive disorders (137). PSD-95 is activated by JNK signaling required in promoting synaptic pruning (138). Also, in patients with HIV infection, studies conducted by positron emission technique have shown that a reduction in synaptic vesicle protein 2A (SV2A) in presynaptic terminals could decrease abnormal density of synapses in the frontostriatal-thalamic circuit, involved in motor and oculomotor function (139). HIV-1 viral proteins, such as Tat, are associated with inflammation, dysregulated neural function, disrupted neurotransmitter systems, and synapse loss through the initiation of complement signaling. There is a special relationship between the complement system and HIV-1 viral proteins mediated axon pruning during brain development (Fig. 2). Thus, the complement cascade can initiate synaptic removal by microglial engulfment during normal development and disease.

HIV-induced synaptic damage is mediated by viral proteins, such as Tat, which disrupt neuronal processes. Also, dysregulation of the NMDA receptor leads to excitotoxicity, mitochondrial dysfunction, and oxidative stress. This direct neuronal damage is further increased by neuroinflammation. Although activation of the complement system may play a secondary role in induced neuronal stress. This distinguishes HIV from other neurotropic viruses and highlights the potential for neuroprotective strategies that stabilize neuronal calcium homeostasis.

HIV-associated synaptic pruning is characterized by a more direct attack, in contrast to the complement mechanisms. The viral proteins Tat and gp120, by over-activating NMDA receptors, destroy neuronal cells, causing high influx of calcium and oxidative stress. In contrast, flaviviruses often exert innate immune-mediated synaptic pruning.

### Rabies virus

Rabies, as a negative-stranded RNA virus, is caused by rabies lyssavirus (140). Rabies virus (RABV) belongs to the family of Rhabdoviridae and affects bats, skunks, foxes, dogs, and humans. In animals, RABV accumulates in salivary glands and spreads to various parts of the body. RABV in the saliva of infected animals can survive for years. In mammals, the RABV affects the CNS. Research has been performed in the field of RABV vectors in synaptic plasticity. It has been indicated that the release of dopamine, GABA, and glutamate in neuroplasticity can be changed in mice infected with RABV. So Ghasemi et al. reported that in the DG of the hippocampus, the induced expression of the Rabies Virus Glycoprotein (RVG) promotes LTP and paired-pulse facilitation as a model of synaptic plasticity (78). Aliakbari et al. reported that RVG can increase hippocampal-dependent memory in the hippocampus in a model of AD (141). Therefore, we found that RVG changes synaptic function and synaptic transmission in both presynaptic and postsynaptic terminals. This synaptic facilitation is associated with the release of neurotransmitters such as glutamate. While our data indicate that RVG enhances synaptic strength, the outcomes might vary significantly with the RABV, given evidence suggesting that it can disrupt synaptic activity. RVG, via switching the IRE1-JNK pathway, plays an essential role in autophagy and apoptosis (142). JNK signaling, a novel pathway involved in pruning of neuronal sensory in Drosophila (132). Few studies have focused on the synaptic elimination caused by rabies. Previous research has shown that morphological alterations occur in dendritic cortical pyramidal neurons with RABV infection (143). Yan Song et al. indicated that a significant decrease in mice infected with RABV occurs in dendritic spines of the CA_1_ region (144). This decrease is partially caused by the depolymerization of actin filaments (F-actin) (144). Li et al. also reported that mice infected with CVS initiated a disorganization in neuronal dendrites in the hippocampus (145). While the neuropathology caused by viruses such as HIV and severe acute respiratory syndrome coronavirus 2 (SARS-CoV-2) relies mainly on indirect mechanisms, including the induction of systemic inflammation and activation of microglia, leading to abnormal synaptic pruning, the RV follows a more direct and aggressive pathogenic pathway. It directly infects neurons by exploiting mechanisms of trans-synaptic spread and causes widespread neuronal loss by inducing apoptosis. This process ultimately leads to the collapse of synaptic networks and the development of fatal clinical manifestations. Thus, despite sharing the outcome of synaptic dysfunction, the RABV pathogenic pathway is unique in its directness and cellular nature (146, 147). These viruses selectively utilize the host’s trans-synaptic spread machinery and molecular motors such as dynein (a motor protein for retrograde transport) to systematically infect neurons and cause cell death by inducing apoptosis. Although dynein does not directly remove synapses, it plays a key role in facilitating the process of synaptic pruning. This role is achieved through the retrograde transport of cargoes essential for synapse health, as well as the anterograde transport of inflammatory signaling molecules in microglia. Dysfunction of dynein can weaken synaptic integrity, making it a target for abnormal pruning by microglia (30).

RABV induces synaptic pathology through a different mechanism that focuses on direct cytotoxicity. It has been shown to destroy dendritic spines and presynaptic structures by degrading cytoskeletal structures (such as F-actin subunits) and triggering apoptotic pathways. Therefore, rabies indicates a “bystander” model of synapse loss in which synapses are destroyed, which distinguishes it from all viruses that are involved in synaptic pruning.

The RABV exhibits a unique pattern of viral-mediated synaptic pathology. Unlike the immune-mediated mechanisms of ZIKV and WNV or the direct targeting of ion channels by viral proteins as seen in HIV, the profound synaptic disruption in rabies results from the virus’s direct action on cytoskeletal proteins, which weakens neuronal connections.

### Coronavirus

The SARS-CoV-2 causes neuronal alterations in the infected brain and impacts the CNS, contributing to neurological disorders, including stroke, hallucinations, headache, anorexia, diarrhea, pain, epilepsy, and encephalopathy (148). Chronic inflammation and production of inflammatory cytokines created by SARS-CoV-2 lead to pathological effects on the brain (149). It is shown that the infection of SARS-CoV-2 in cortical neurons leads to an imbalance in the ion channel function and decreases the number of excitatory synapses and pre-synaptic proteins, associated with neuronal death (150). Reduction of excitatory synapses can be considered as a stage of synaptic pruning. Acute SARS-CoV-2, through interaction with Toll-like receptor 4 (TLR4), triggers synaptic pruning and memory dysfunction in the hippocampus of mice with COVID-19 syndrome (151). TLR4 activates NF-κB and subsequently secretes inflammatory cytokines such as IL-1 and TNF-α, leading to the conversion of C1q into C3, which promotes pruning of hippocampal synapses (152, 153). Blocking TLR4 signaling, either genetically or pharmacologically, safeguards animals from synapse loss and memory impairment caused by spike brain infusion (151). Furthermore, several imaging studies suggested that COVID-19 exposure triggered a decrease in dendritic spines associated with synaptic pruning (154). Brain scans of COVID-19 patients have demonstrated that following COVID-19 infection, changes in the hippocampal volume and cognitive function impairment (155). An investigation of the patient’s brain infected by SARS-CoV-2 demonstrated that this virus exacerbated the pruning of synapses and synapse engulfment (84). However, these changes were not demonstrated at the early stage of synaptic density (151). Studies on brain tissue from patients who died from severe COVID-19 have shown inflammation and neuronal damage, which is linked to synapse loss. Microvascular damage and inflammation in the brains of COVID-19 patients show that widespread inflammation (glial reaction) is a proven factor in synapse loss (156). Findings from human brain tissue indicate that SARS-CoV-2 infection leads to impaired myelination and neuronal unsheathing. Since myelin integrity is critical for efficient transmission of synaptic signals, these results could be evidence of impaired function and possibly pruning of synapses due to viral infection (157). SARS-CoV-2 also promotes microglia-mediated phagocytosis and increases the high level of genes involved in the elimination of synapses such as Cluster of Differentiation 68 (CD68), TREM2, Integrin Subunit Beta 5 (ITGB5), Cluster of Differentiation 47 (CD47), Macrophage Scavenger Receptor 1 (MSR1), and Calreticulin Receptor (CALR) in patients recovering from COVID-19 (84). CD47, through binding to signal regulatory protein alpha (SIRP), protects synapses from excessive elimination in the CA_1_ region of the hippocampus. Although overexpression of SIRP through the decrease of PSD-95 decreased dendritic density (158). PSD-95-modulated endocytosis of NR2A-containing NMDARs (a subunit of NMDA receptor) (159) is involved in synapse turnover in the cerebral cortex (160). Together, these results suggest that SARS-CoV-2 activation mediates synaptic pruning induced by microglia in mice. It has been reported that there is a direct relationship between SIRP and JAK/STAT in the CNS (161). SIRPα also interacts with CD47 in both human AD patients and AD mice, which contributes to the loss of synapses (22). This pathway plays a fundamental role in synaptic pruning. However, the role of TLR4 in LPS-induced microglia activation in synaptic pruning is unclear. Recently, it has been reported that APOE in neurons infected by SARS-CoV-2 leads to a reduction in dendritic length and synaptic loss (162). In APOE4 mice, this process occurs due to heightened levels of three cytokines (IL-1β, IL-6, and TNF-α) in intracerebroventricular (163). These findings proposed that SARS-CoV-2 is involved in excessive synaptic pruning through TLR4 signaling. There is a noticeable relation between the tyrosine kinase downstream signaling pathway and SARS-CoV-2 that may be caused by the large number of proinflammatory cytokines and chemokines. For example, JAK2 signaling plays a key role in the elimination of inactive synapses in multiple brain regions, such as in the cingulate cortex via significant increases of Synaptophysin and VGLUT_1_ (164). It is indicated that when the Suppressor of Cytokine Signaling 3 (SOCS3) is absent in nerve cells, the JAK2 signaling pathway goes out of control and becomes overactive. This excessive activation, in turn, causes the process of synapse elimination (both physical and functional) to occur with much greater intensity and scope (164). Di Liberto et al. reported that JAK/STAT can activate proinflammatory genes, chemokines, and cytokines in neurons involved in activating microglia to complete synapse elimination (165). Also, it is indicated that infection with SARS-CoV-2 induces a cytokine storm (e.g., IL-6, IFN). These cytokines reach the brain and activate the JAK/STAT pathway in microglia. Sustained activation of this pathway shifts microglia into an aggressive state that leads to abnormal synaptic pruning in three ways: (i) Synapse marking: synapses are marked for destruction by increased production of C1q and C3 (from the complement system). (ii) Synapse engulfment: By increasing receptors such as CR3 on microglia themselves, which allows them to engulf marked synapses. (iii) Perpetuation of inflammation: By creating a vicious cycle of further cytokine secretion, leading to ongoing damage. The JAK/STAT pathway is a molecular link between viral infection and brain damage that explains the symptoms of “brain fog” in long-term COVID. Inhibition of this pathway (with JAK inhibitor drugs) is a promising therapeutic target (166, 167).

Thus, COVID-19 infection in the brain may be involved in functional and structural synapse elimination via inflammatory cytokines, tyrosine kinase signaling, and chemokines (Fig. 2).

SARS-CoV-2-increased synaptic pruning displays a powerful interplay between innate immune and microglial activation. The viral spike protein, via the TLR4 receptor, triggers a strong inflammatory response characterized by activation of the NF-κB signaling pathway, complement system (C1q/C3) signaling, and the JAK/STAT pathway. This mechanism highlights a pathogenic axis that may help demonstrate the role of viral infections in chronic neurological diseases.

Activation of the JAK/STAT signaling in microglial cells, which causes a vicious cycle of inflammation and synaptic dysfunction and likely underlies cognitive symptoms in long-term COVID, is a feature that has been less prominent in other neurotropic infections.

### Influenza A virus

Influenza virus, as a segmented negative-sense single-stranded RNA segment, is a member of the family Orthomyxoviridae. Four types of this family have been identified: types A, B, C, and D. It has been reported that type B occurs in humans, while type A only infects pigs and horses. This virus, with a high risk of death worldwide, has been reported to affect animals such as birds and mammals (168). The influenza virus mainly affects the nose, throat, bronchi, and lungs and is easily transmitted from person to person via small particles when infected people cough or sneeze. In addition to the lungs, in clinical investigations, several studies indicated that influenza A has been shown to create myocarditis, lung disease, metabolic disorders such as diabetes, and neurodegenerative diseases, such as febrile seizures, focal encephalitis, narcolepsy, major depression, and PD-like symptoms (169, 170). It is reported that 4 days after the induction of inflammation via influenza, this virus induces neuroinflammation through altered proinflammatory/inflammatory cytokine expression, including IL-1 and TNF, in the CA_1_ (171), affecting several biological functions such as fear, learning, and memory processes. In the CA_1_ and DG of the hippocampus of mice infected by this virus, pro-inflammatory markers such as IL-1, IL-6, and TNF-α elevate neuroinflammation (172). These findings suggest that during influenza infection, an alteration in hippocampal plasticity may be associated with cognitive impairment. It has been suggested that the influenza virus can change neuronal plasticity in the hippocampus and amygdala complex. However, these mechanisms in the CNS are not understood. For instance, the acute influenza virus can change hippocampal dendritic spine density, thereby influencing synaptic plasticity and structure through a decrease in neurotrophic factor signaling and immunomodulatory factors (173), which are critical for synaptic plasticity and neuronal survival in the brain. Therefore, the influenza virus can alter the morphology of hippocampal neurons, including CA_1_ pyramidal neurons and DG granule cells (DGCs), which are associated with synaptic pruning in the brain. Consequently, the influenza virus increases hippocampal inflammation and causes significant alterations in neuron morphology in the hippocampal formation. Jurgens et al. indicated that the influenza virus caused cognitive dysfunction created by changes in neuron morphology in adult mice (172). Loss of the dendritic spine can induce functional and structural alterations in hippocampal formations associated with a reduction in LTP. In addition, neurotropic strains of influenza, such as H7N7 and a non-neurotropic H3N2 infection, caused spine loss and long-term impairments in the brain. Although acute infection with the H1N1 virus impacts some dendritic spines, it does not result in significant long-term effects (173). It was shown that infection with a low-dose non-neurotrophic H1N1 infection, in the cortex and hippocampus, resulted in synaptic pruning alterations that occurred through the activation of microglia (80). Overall, infection with the Influenza A virus appears to reshape neuronal synapses and function through microglia activation associated with neuroinflammation (Fig. 2). Previous work has indicated that mice infected with H1N1 exhibited a significant alteration in neuron morphology, such as a significant loss of distal dendrites in the DG (172). This virus can also augment proinflammatory and anti-viral mediators such as IL-1β in the mouse hippocampus, correlated to synaptic elimination (172). *In vitro* studies indicated that IL-1β reduced the density of dendritic spines in the cerebral cortex (174). Also, IL-1β leads to changes in the arrangement of hippocampal synapses (175). When IL-1β binds to the IL-1R1 receptor on neurons, it activates the following signaling cascade: MyD88, IRAK, and TRAF6, NF-κB, and p38 MAPK, resulting in a decrease in the expression of synaptic proteins such as PSD95 and a decrease in AMPA (channels permeable to Na^+^ and Ca2+) and NMDA expression (176). The NF-κB and p38 MAPK signaling play a major role in synaptic pruning and are activated by the influenza virus (177). Although direct human neuropathological evidence for influenza-induced synaptic loss is scarce, robust epidemiological studies have linked influenza infection to an increased risk of neurodevelopmental disorders and PD later in life. However, animal models suggest that systemic inflammation and subsequent neuroinflammation induced by influenza infection can lead to microglial activation and abnormal synaptic pruning, particularly during vulnerable developmental windows. This mechanism is hypothesized to underlie the long-term neurological sequelae observed in some patients. Thus, influenza virus infection can impact functional and structural alterations in hippocampal networks through inflammatory factors.

The influenza A virus drives synaptic loss through both direct and indirect mechanisms. Notably, peripheral infection triggers a proinflammatory cytokine response (e.g., IL-1β, TNF-α), which can enter the CNS and activate microglia. In the hippocampus, these activated microglia engage in excessive synaptic pruning, reducing spine density and disrupting synaptic plasticity. This demonstrates that direct neuronal infection is not necessary for synaptic damage; a robust immune response alone is sufficient to disrupt CNS connectivity and impair cognitive function. Unlike viruses such as ZIKV or HIV, which directly infect neurons, the influenza virus primarily affects CNS synapses by triggering systemic inflammation that activates microglia.

### Borna disease virus

Borna disease virus (BDV), a non-segmented and negative-stranded RNA genome, implicates a wide range of animals such as domestic mammals and humans (178). Previous studies demonstrated that this virus plays an important role in mental disorders, including unipolar disorder, bipolar disorders, autism, and neuronal dysfunction through targeting neurons of the limbic system (179, 180). Although direct human neuropathological evidence for BoDV-1-induced synaptic pruning is limited. Strong data from animal models demonstrate that BDV plays a major role in synaptic density and neuronal function (181), probably via PKC signaling. BDV in the cortex and hippocampus can serve as a PKC kinase substrate, resulting in the diminished phosphorylation of other PKC neuronal targets, such as Myristoylated Alanine-Rich C-Kinase Substrate (MARCKS), Synaptosomal-Associated Protein 25 (SNAP-25), and Mammalian uncoordinated**-**18 (Munc18), which play important roles in vesicular cycling (81). Several studies have indicated that BV impairs signaling pathways crucial for neuronal functioning and communication via PKC (180, 182). PKC, including PKCα, PKCβ, and PKCγ, is responsible for synapse elimination and synaptic plasticity in cortical neurons (182). Interestingly, knockout of PKCγ results in an impairment in the synaptic elimination in the cerebellum (183). Additionally, the PKCα knockout results in impaired elimination of climbing fibers, albeit to a lesser extent (183). *In vivo* studies have shown that activation of PKC in synaptic pruning occurs during 6–10 days of postnatal development (184). However, Lanuza et al. indicated that PKC is required in the synapse elimination stages triggered 2 weeks after birth at the neuromuscular junction (185). It is reported that infection of acute BDV with interference in PKC signaling impairs synaptic plasticity in cortical neurons (182). In addition, previous studies showed that at the neuromuscular junction, activation of PKC through postsynaptic acetylcholine receptors (AChRs) caused a reduction in postsynaptic receptors and synaptic plasticity (186). Thus, it is possible that BV, via a disturbance in the PKC pathway, causes the elimination of synapses. Additionally, it has been reported that a mutation in PKCγ in Purkinje cells can disrupt the pruning of synapses in spinocerebellar ataxia (187). Synaptic pruning is an event that is associated with both LTP and LTD at the synaptic level (188). LTP promotes a long-lasting increase in neuronal transmissions (189). In contrast, LTD, a process of synaptic weakening, reduces the efficacy of neuronal synapses, resulting in spine shrinkage and loss from dendritic spines in hippocampal slice cultures (190, 191). Also, it has been displayed that inhibition of Metabotropic glutamate receptor 1 (mGluR_1_) impaired the loss of climbing fibers (CFs) synapses in the cerebellum, involved in LTD (192). mGluR1, a G-protein-coupled receptor, plays an essential role in LTP and LTD in the hippocampus (193). It is indicated that following acute infection in neonatal rats in the DG of the hippocampus, BVD can activate caspase-3 (194) and induce LTD in different regions of the brain, such as the hippocampus. Impairment of LTD prevents synaptic pruning and increases dendritic spines in hippocampal neurons (195, 196).

Overall, BVD interferes with PKC signaling largely indirectly through disruption of calcium dynamics, altered host gene expression, and impaired synaptic processes rather than by directly binding or inactivating PKC. The evidence points to a role in synaptic dysfunction and plasticity deficits, but definitive mechanistic proof is still lacking.

BDV impairs neuronal signaling and synaptic function through multiple mechanisms. It disrupts PKC activity, alters growth and pruning processes, and inhibits LTD, thereby preventing the normal elimination of redundant synapses. Furthermore, BDV infection can directly destabilize synaptic connectivity independently of widespread neuroinflammation. This highlights a key distinction from other neurotropic viruses: BDV’s primary strategy is to disrupt synaptic pruning by targeting intracellular signaling cascades, specifically PKC.

### Herpes simplex virus

Herpes simplex virus type 1 (HSV-1), as a neurotropic virus, retrogradely transfers from the axons of the sensory neurons to the CNS and infects over 60% of the population worldwide (197, 198). It has been shown that HSV-1 infection is related to neurodegenerative disorders such as meningoencephalitis (199) and AD (200) through changes in protein expression, axonal cytoskeleton, and cellular homeostasis (201, 202). However, the effect of HSV-1 infection on the structure or function of the spines and dendritic arborization is unknown. It has been indicated that acute HSV-1 infection enhances high expression of Arc (Activity-Regulated Cytoskeleton-associated protein) protein related to synaptic plasticity through altered glutamatergic responses and decreased PSD-95, Drebrin (DBN), and Ca^2+^/calmodulin-dependent protein kinase II beta (CaMKIIb) expression in cultured cortical neurons (82). Arc protein, an immediate-early gene, mediates the internalization of glutamate receptors and is essential for synaptic scaling. The Arc protein in the cerebellum regulates the elimination of the climbing axons to Purkinje cells (203). This reduction in infected neurons is associated with a decrease in dendritic arborization (82). Acuna et al. reported that during acute infection of HSV-1, the expression of Arc protein increased in neuronal cell lines (204). The Arc protein plays a crucial role in memory formation by interacting with protein complexes, including PSD-95, Drebrin, and CaMKIIb. This protein targets inactive dendritic spines through the interaction with CaMKIIb (82, 204). CaMKIIb is a subunit of the enzyme CaMKII, a key calcium sensor in neurons, which plays a crucial role in synaptic plasticity, learning, and memory. The activation of Ca^2+^/CaM-dependent enzymes, such as CaMKII and PKA, suppresses attraction to repulsion in vertebrates (205). Finally, CamKII activates an Eag/CASK-dependent mechanism (Ether-à-go-go/calcium [Ca^2+^])/calmodulin- (108) associated serine kinase) required in axon terminal growth (206). In this regard, Okuno et al. also reported that the Arc protein is recruited to unnecessary spines in the CA_1_ (207). In connection with the key role of this virus in synaptic plasticity, it has been indicated that during acute HSV-1 infection, cAMP response element binding protein (CREB) activation induced Arc protein expression in Vero cells (208), which was associated with LTP. CREB is a key transcription factor that regulates the expression of genes essential for memory formation and long-term synaptic plasticity (209). In addition, it is shown that chronic infection with HSV-1 also promotes calcium signaling in rat cortical neurons with modified synaptic morphology in AD (210). Intracellular calcium levels are involved in neurite formation and gene transcription, which modulate synaptic plasticity and synaptic pruning in various brain regions (Fig. 2). Previous studies have declared that inflammation plays a major role in LTP, LTD, and synaptic pruning (211). During acute HSV-1 neuronal infection, inflammatory mediators such as IFN-β, IFN-γ, IL-1β, IL-6, chemokine (C–X–C motif) ligand 8 (CXCL8), TNF-α, and TGF-β produced by neurons and glial cells increase in the bed nucleus of the stria terminalis, medial amygdala, accessory OB, and ventromedial hypothalamus (212), which contributed to the loss of synaptic density (213, 214). Recently, some studies have indicated that after HSV infection, microglia release IL-1β (215, 216). IL-1β creates synaptic scaling in the cerebellum through the mammalian target of rapamycin (mTOR) signaling in mice with autistic-like behaviors (217). Also, it has been reported that a lack of IL-1 receptor displays an increase in dendritic spines in the DG of the hippocampus, such as hilus and granular cell layer (GCL) in mice (218). Additionally, receptor accessory protein (IL-1RAcP), a cell adhesion molecule in neurons, induces synaptic changes in newborn hippocampal neurons during synapse formation in neuroinflammation (219). Although primarily conducted in a mouse model, it also provides data from human tissue that suggests that acute and chronic HSV-1 infection is associated with markers of neuropathology (such as abnormal Tau protein aggregation) that can lead to synaptic dysfunction or pruning (220). Therefore, the interaction of inflammatory cytokines and HSV infection can play an essential role in synaptic elimination.

HSV-1 induces synaptic damage through two mechanisms. First, it directly upregulates Arc, causing significant changes in the synaptic proteome that result in the internalization of glutamate receptors and the loss of vital structural components such as PSD-95, which destroys synapses. Second, it induces a neuroinflammatory response, with cytokines such as IL-1β further destabilizing synaptic integrity. Thus, intrinsic neuronal disruption and extrinsic immune activation remarkably allow HSV-1 to remodel neural circuits and contribute to its correlation with neurodegenerative diseases.

HSV-1 employs a unique, two-pronged strategy for synaptic modulation. It shares a direct mechanism with RABV by targeting synaptic proteins like Arc to disrupt neuronal integrity. Simultaneously, it shares an indirect mechanism with viruses like ZIKV and WNV by driving a sustained neuroinflammatory response that mediates pruning. This hybrid approach—direct viral sabotage combined with relentless host immune attack—distinguishes HSV-1’s pathogenesis from the more specialized strategies of the other neurotropic viruses mentioned.

## COMPARATIVE MECHANISMS OF VIRAL MODULATION OF SYNAPTIC PRUNING

**Fig 3:**
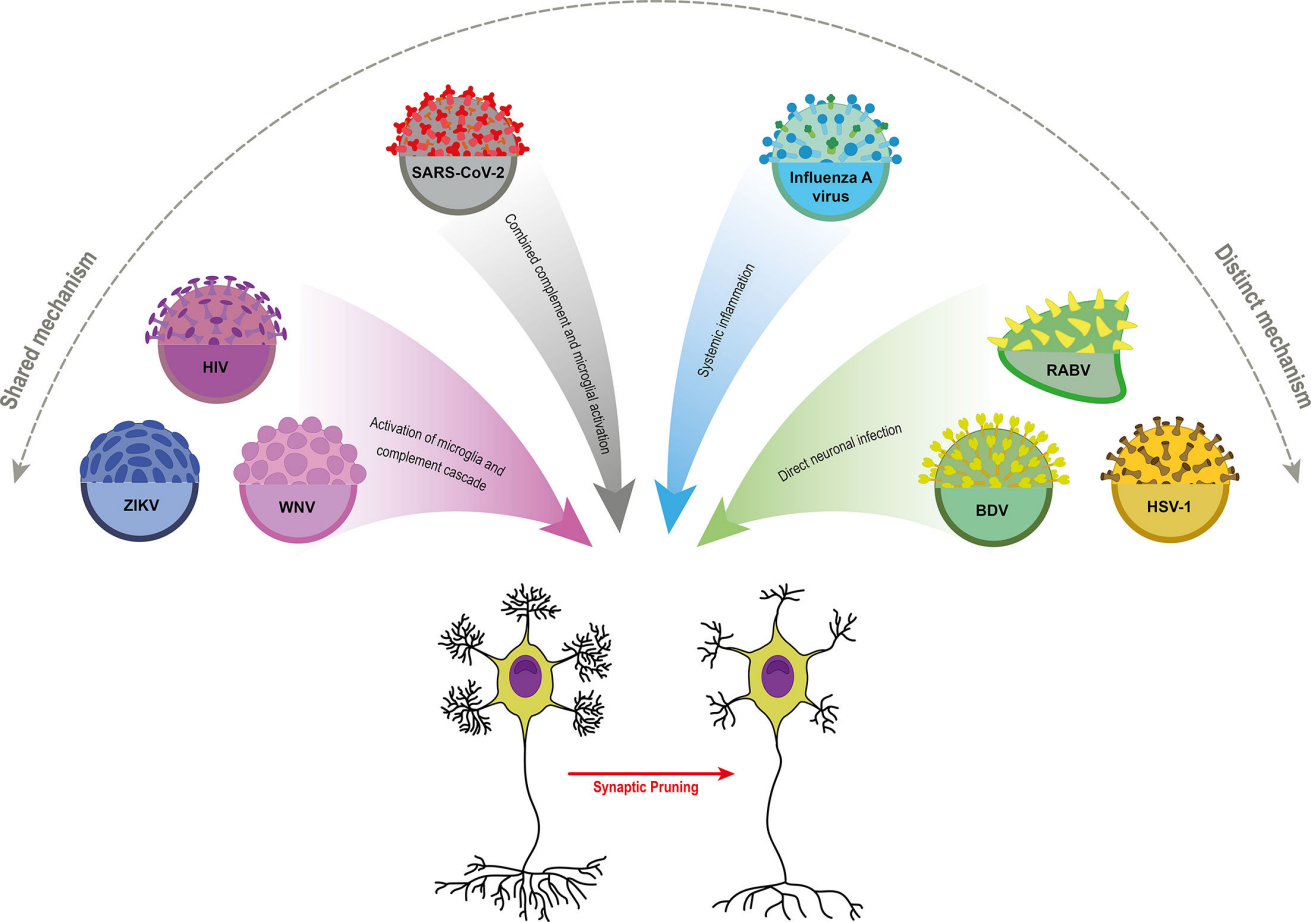
Viral-induced synaptic pruning involves both common and distinct pathways. WNV, HIV, and ZIKV utilize a shared mechanism by activating microglia and the complement system, leading to synaptic elimination. In contrast, viruses such as RABV, Borna virus (BV), and HSV trigger synaptic loss through more direct mechanisms, including systemic effects and direct neuronal alterations.

Various neurotropic viruses have evolved distinct strategies to disrupt synaptic communication, often by recruiting specific host or viral proteins that interfere with neurotransmission and plasticity (Table 2). While the outcome may be similar, such as the excessive pruning of synapses seen in cognitive decline, the underlying mechanisms can vary significantly. Some pathogens may trigger shared, non-specific neuroinflammatory pathways that lead to synaptic loss, whereas others employ highly specific and distinct molecular pathways, such as directly cleaving synaptic structural molecules or hijacking complement-mediated pruning pathways typically used in brain development (Fig. 3).

**TABLE 2:** Viral proteins, or host proteins recruited by viruses, cause synaptic dysfunction or elimination

Protein	Viral infection	Signaling pathway
Nrf2	ZIKV	GSH, GCLC, and G6PD (86)
IFN-**γ**	ZIKV	JAK1/JAK2/STAT (96)
Tat	HIV	Ca^2+^/GluN2B signaling and PERK/eIF2α (protein kinase RNA-like ER kinase/Eukaryotic Initiation Factor 2 alpha) pathway (125) and NMDA signaling (77)
gp120	HIV	Wnt/β–catenin/FKN/CX3R1 pathway (130)
PSD-95	HIV, HSV, and COVID	Internalization of NR2A-containing NMDARs (159), through Erk-independent signaling (82) and JNK signaling (138)
PKC	BDV	Via mTORC2-AKT-GSK3β, it internalizes AMPA and increases LTD (196)
Arc	HSV	CaMKIIb/Debrin complex (221)
IL-1β	ZIKV, COVID, and HSV	mTOR signaling (217) and NF-κB and p38 MAPK signaling (177)
TNF-α	WNV, COVID, influenza, and HSV	C1q/C3e caspase-8/caspase-3 (112)
F-actin	RABV	Depolymerization of actin filaments (144) and disorganization in apical dendrites (145)

### Shared mechanisms

The activation of both microglia and the complement cascade represents a common pathogenic mechanism observed in numerous viral infections. For instance, WNV, HIV, and ZIKV infections promote complement component (C1q, C3) deposition on synapses, facilitating microglial engulfment. In both cases, viral-induced neuroinflammation acts as a critical driver, with elevated cytokines (e.g., IL-1β, TNF-α) reinforcing pruning pathways. This suggests that immune-mediated synaptic clearance represents a convergent mechanism by which diverse viruses impact neural circuits.

### Distinct mechanisms

Despite these shared pathways, several viruses exploit unique processes. RABV and HSV can directly infect neurons, altering synaptic protein expression and cytoskeletal organization, thereby increasing synaptic vulnerability to pruning. Through a mechanism similar to PKC chelation, BV impairs synaptic function and promotes synaptic pruning. Influenza virus, although not typically neurotropic, influences pruning through activation of systemic immune and cytokine release, which secondarily modulate microglial activity in the CNS. Meanwhile, SARS-CoV-2 has been linked to dysregulation of astrocytic and microglial signaling, suggesting a glia–virus interaction distinct from the classical complement pathway.

### Integrative perspective

Taken together, these findings highlight that while viral infections often converge on immune-mediated mechanisms of synaptic pruning, the precise molecular routes and cellular targets can differ markedly. Some viruses act primarily through direct neuronal disruption (e.g., RABV, BV, and HSV), others through systemic inflammation (e.g., influenza), and yet others through a combination of complement and microglial activation (e.g., HIV, SARS-CoV-2, and Zika). Recognizing both these overlapping and divergent processes provides a framework for understanding how viral infections differentially sculpt synaptic connectivity, which may help explain the variability in cognitive and behavioral outcomes observed across viral neuropathologies.

## CONCLUSION

In conclusion, synaptic pruning is an essential neurodevelopmental process that plays a critical role in optimizing neural circuits and overall brain function. This process is vital for cognitive development, learning, memory consolidation, and maintaining brain health. However, viral infections pose a significant threat to the integrity of synaptic pruning. These infections can disrupt synaptic plasticity, protein homeostasis, and the delicate balance of synaptic elimination and spine pruning. The alterations caused by viral infections, such as changes in spine protein expression, activation of the complement system, and the release of inflammatory markers, can lead to either excessive or insufficient synaptic pruning. These disruptions have profound neuropathological consequences, including cognitive impairments and the potential onset of neurodegenerative diseases. Our review highlights the need for a deeper understanding of the mechanisms through which viral infections impact synaptic pruning. By unraveling these complex interactions, we can pave the way for future research and therapeutic interventions aimed at mitigating the adverse effects of infections on brain function. Ultimately, this knowledge will contribute to the development of strategies to preserve and enhance neural health in the face of infectious challenges.

## PERSPECTIVE AND FUTURE DIRECTIONS

The connection between viral infections and synaptic pruning opens up an exciting yet complex area of neuroscience. Synaptic pruning is essential for shaping healthy brain circuits, but when viruses interfere with this process, the results can be far-reaching—from cognitive decline to developmental disorders. As growing evidence shows, viruses can disrupt the delicate balance of synaptic refinement by triggering immune responses, altering complement activity, and changing the expression of key synaptic proteins. Understanding how these processes intertwine could transform how we think about both brain development and the long-term impact of infections on neural health.

Looking ahead, it is important to view viral effects on synaptic pruning as part of a broader conversation between the nervous and immune systems. The brain is not an isolated organ—it constantly interacts with immune signals, and infections can leave lasting marks on this communication. Investigating how viruses influence microglia and astrocytes, the brain’s key immune cells, may reveal how these cells shift from supporting healthy pruning to contributing to synaptic damage.

Future studies should move in three main directions. First, long-term imaging and molecular studies are needed to track how viral infections change synaptic networks over time and whether those changes can be reversed. Second, mechanistic research should identify which viral and host molecules disrupt normal pruning pathways, especially those involving the complement system and immune signaling. Third, therapeutic research should explore ways to fine-tune the immune response or block harmful viral effects to restore normal synaptic function.

By bridging the fields of neuroimmunology, virology, and neuroscience, researchers can gain a clearer picture of how infections shape brain connectivity. This integrated approach could lead not only to a better understanding of virus-related neurological conditions but also to new strategies that protect synaptic health and preserve cognitive function throughout life.

## Data Availability

Data sharing does not apply to this article, as no new data were created or analyzed in this study.
